# Age and APOEε4 correlate with cognitive improvement after Lecanemab treatment.

**DOI:** 10.1002/alz70859_104146

**Published:** 2025-12-25

**Authors:** Lin Kang, Yulei Deng, Binyin Li, Yingting Zheng, Xinyuan Yang

**Affiliations:** ^1^ Ruijin Hospital Affiliated to The Shanghai Jiao Tong University Medical School, Shang Hai, Shang Hai China; ^2^ Ruijin Hospital affiliated with Shanghai Jiao Tong University School of Medicine, Shanghai, Shanghai China; ^3^ Ruijin Hospital affiliated to Shanghai Jiaotong University School of Medicine, Shanghai, Shanghai China

## Abstract

**Background:**

There have been insufficient data for safety and efficacy of Lecanemab therapy. Our research aimed to determine which character of the patients are associated with efficacy for AD patients treated with Lecanemab.

**Method:**

The single‐arm, open‐label trial was designed to evaluate the effects of biweekly lecanemab administration over 26 weeks in 26 patients aged 56 to 81 years. The primary endpoint was the change from baseline at 6 months in the Mini‐Mental State Examination (MMSE) score. Secondary outcomes included plasma Aβ42, Aβ4, GFAP, NfL, pTau181, pTau217, HAMD, HAMA, and CDR‐SB.

**Result:**

The mean change in MMSE from baseline (23.30) to 6 months (22.34) was −0.96 (95% confidence interval [CI], −2.10 to −0.28; P = 0.122). Other mean differences in pre‐therapy and post‐treatment were as follows: Aβ42, 1.93 (95% CI, 1.03 to 2.84; P < 0.001); Aβ40, 46.46 (95% CI, 28.30 to 64.62; P < 0.001); pTau181, −1.21 (95% CI, −1.81 to −0.61; P < 0.001); NfL, 4.98 (95% CI, 0.21 to 9.74; P < 0.05); HAMD, 0.462 (95% CI, −0.694 to 1.617; P = 0.418); HAMA, −0.557 (95% CI, −3.26 to −0.94; P = 0.37); and CDR‐SB, −0.12 (95% CI, −0.73 to −0.84; P = 0.70). Furthermore, the improvement in MMSE scores was significantly negatively correlated with age (r = −0.558, p < 0.01). Additionally, a significant difference in MMSE score improvement was observed between patients aged above 75 (*n* = 19) and those aged 75 or below (*n* = 7), as well as between APOE carriers (*n* = 16) and non‐carriers (*n* = 10) (p < 0.05). In logistic regression analysis, gender and APOE carrier status were not significantly associated with MMSE score change, while age remained significantly negatively associated (R² = 0.35, β = −0.27, p < 0.01). Lecanemab resulted in adverse reactions in 30.77% of the participants.

**Conclusion:**

Although no significant cognitive score improvement was observed in the 26 Chinese patients after six months of treatment with lecanemab, improvements were seen in plasma biomarkers pTau181 and NfL. Plasma Aβ42 and Aβ40 significantly increased, suggesting altered Aβ protein metabolism through the bloodstream. Age and APOEε4 carrier status impacted cognitive improvement.

